# YAP and TAZ Heterogeneity in Primary Liver Cancer: An Analysis of Its Prognostic and Diagnostic Role

**DOI:** 10.3390/ijms20030638

**Published:** 2019-02-01

**Authors:** Matthias Van Haele, Iván M. Moya, Ruçhan Karaman, Guy Rens, Janne Snoeck, Olivier Govaere, Frederik Nevens, Chris Verslype, Baki Topal, Diethard Monbaliu, Georg Halder, Tania Roskams

**Affiliations:** 1 Department of Imaging and Pathology, Translational Cell and Tissue Research, KU Leuven and University Hospitals Leuven, 3000 Leuven, Belgium; janne.snoeck@student.kuleuven.be (J.S.); Tania.roskams@uzleuven.be (T.R.); 2 Facultad de Ingeniería y Ciencias Aplicadas, Universidad de Las Americas, Quito 170503, Ecuador; ivan.moya@kuleuven.vib.be; 3 VIB Center for Cancer Biology, and KU Leuven Department of Oncology, University of Leuven, 3000 Leuven, Belgium; ruchan.karaman@kuleuven.vib.be (R.K.); georg.halder@kuleuven.vib.be (G.H.); 4 Motor Control Laboratory, Movement Control and Neuroplasticity Research Group, Department of Kinesiology, KU Leuven, 3000 Leuven, Belgium; guy.rens@kuleuven.be; 5 Institute of Cellular Medicine, Newcastle University, Newcastle upon Tyne 3000, UK; Olivier.Govaere@newcastle.ac.uk; 6 Department of Hepatology, KU Leuven and University Hospitals Leuven, 3000 Leuven, Belgium; Frederik.nevens@uzleuven.be (F.N.); Chris.verslype@uzleuven.be (C.V.); 7 Department of Abdominal Surgery, KU Leuven and University Hospitals Leuven, 3000 Leuven, Belgium; Baki.topal@uzleuven.be; 8 Abdominal Transplantation Surgery, KU Leuven and University Hospitals Leuven, 3000 Leuven, Belgium; diethard.monbaliu@uzleuven.be

**Keywords:** HCC, cHCC-CCA, CCA, YAP, TAZ, Keratin 19, Hepatic progenitor cells

## Abstract

Primary liver cancer comprises a diverse group of liver tumors. The heterogeneity of these tumors is seen as one of the obstacles to finding an effective therapy. The Hippo pathway, with its downstream transcriptional co-activator Yes-associated protein (YAP) and transcriptional co-activator with PDZ-binding motif (TAZ), has a decisive role in the carcinogenesis of primary liver cancer. Therefore, we examined the expression pattern of YAP and TAZ in 141 patients with hepatocellular carcinoma keratin 19 positive (HCC K19^+^), hepatocellular carcinoma keratin 19 negative (HCC K19^−^), combined hepatocellular–cholangiocarcinoma carcinoma (cHCC-CCA), or cholangiocarcinoma (CCA). All cHCC-CCA and CCA patients showed high expression levels for YAP and TAZ, while only some patients of the HCC group were positive. Notably, we found that a histoscore of both markers is useful in the challenging diagnosis of cHCC-CCA. In addition, positivity for YAP and TAZ was observed in the hepatocellular and cholangiocellular components of cHCC-CCA, which suggests a single cell origin in cHCC-CCA. Within the K19^−^ HCC group, our results demonstrate that the expression of YAP is a statistically significant predictor of poor prognosis when observed in the cytoplasm. Nuclear expression of TAZ is an even more specific and independent predictor of poor disease-free survival and overall survival of K19^−^ HCC patients. Our results thus identify different levels of YAP/TAZ expression in various liver cancers that can be used for diagnostics.

## 1. Introduction

Hepatocellular carcinoma (HCC) incidence rates have been increasing for the last couple of decades [1]. Primary liver cancer (PLC) is the second most common cause of cancer-related deaths worldwide. The most frequent risk factors worldwide are hepatitis B virus (HBV) and chronic hepatitis C virus (HCV) infection, which lead up to cirrhosis and, eventually, HCC. Furthermore, the prevalence of non-alcoholic fatty liver disease (NAFLD) is increasing dramatically and is known to be an important risk factor for HCC [2,3]. It is suggested that NAFLD may soon overtake HBV and HCV as most common etiology of HCC [4]. However, the majority of studies into the carcinogenesis of HCC use cohorts of patients that are dominated by a viral-induced liver disease in the background, while other etiologies are not sufficiently represented.

A very fascinating group of PLC is the combined (or mixed) hepatocellular–cholangiocarcinoma (cHCC-CCA), which presents mixed characteristics of HCC and cholangiocarcinoma (CCA) in one tumor. This dual morphology and recent molecular findings suggest that the cell of origin of these tumors is the hepatic progenitor cell (HPC) [5,6,7]. However, despite many decades of research on cHCC-CCA, there is a marked lack of a consensus in the terminology used to describe these tumors. This is due to a high degree of intratumoral heterogeneity in cHCC-CCA tumors [8]. For this reason, an international platform of experts has agreed upon a pathological description of this tumor subgroup. The internationally accepted description of cHCC-CCA provides the opportunity for more in-depth investigation of these rare tumors of possible HPC-derived origin [8]. In addition, we and others have demonstrated the existence of a subgroup of keratin 19 positive (K19^+^) HCCs within the group of HCCs. These K19^+^ HCCs are more aggressive than keratin 19 negative (K19^−^) HCCs [9,10,11,12,13]. Therefore, liver cancer is a highly heterogeneous disease, and understanding the molecular pathways governing this heterogeneity is crucial for the development of effective therapeutic approaches against the different subtypes of liver cancer [14].

The Hippo signaling pathway is a signal transduction pathway that plays important roles in the regulation of organ growth, cell proliferation, and cellular plasticity [15,16,17]. In recent years, the Hippo pathway has attracted a lot of attention because it is deregulated in most human cancers and can trigger tumorigenesis in experimental mouse models [18,19]. The core of the Hippo pathway comprises a kinase cascade of the STE20-like protein kinases (MST1/2), and the Large tumor suppressor kinases (LATS1/2), which phosphorylate and repress the activity of the transcriptional co-activators Yes-associated protein 1 (YAP) and its homolog WW domain-containing transcription regulator protein 1 (WWTR1, also called TAZ) [20]. Phosphorylated YAP and TAZ are retained in the cytoplasm and targeted for proteasomal degradation, while non-phosphorylated YAP/TAZ can enter the nucleus and bind to the TEA Domain Transcription Factors (TEAD 1–4) to regulate target gene expression [21]. Although YAP and TAZ play highly redundant roles, there is some emerging evidence suggesting that they might have different functions in primary liver cancer [22]. However, little is known about their function in primary liver cancer and how they contribute to the development of the different subtypes of liver tumors. Thus, we aimed to investigate the expression of YAP and TAZ in a cohort of Caucasian primary liver patients in a mainly non-viral setting. We explored YAP and TAZ with interest in their subcellular localization and prognostic significance. 

## 2. Results

### 2.1. General Clinicopathological Characteristics of HCC, cHCC-CCA, and CCA Patients

**Figure 1:**
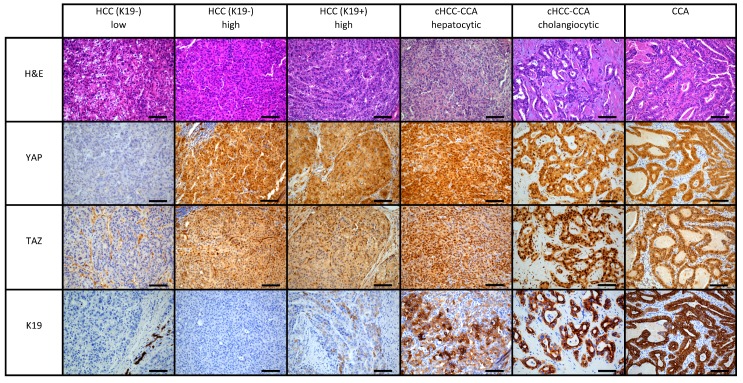
Representative immunohistochemistry images of YAP, TAZ, Keratin 19 (K19) is shown for hepatocellular carcinoma (HCC) K19^−^, HCC K19^+^, combined hepatocellular–cholangiocarcinoma (cHCC-CCA), and cholangiocarcinoma (CCA) cases. The spectrum of HCC Keratin 19^−^ is illustrated by a case that shows the absence of YAP and TAZ in the tumor cells, while another case of HCC Keratin 19^−^ illustrates high expression of YAP and TAZ. HCC K19^+^ is represented by a case that has high YAP and TAZ expression. The hepatocellular and cholangiocellular components show expression in the cytoplasm and nucleus. CCA shows positivity for YAP and TAZ. All images were taken at 20×. Scale bar: 100 µm.

For our studies, we analyzed a cohort of PLC patients treated at the University Hospitals of Leuven. This cohort included 81 K19^−^ HCC, 13 K19^+^ HCC, 35 cHCC-CCA, and 12 CCA patients. First, we defined the clinicopathological characteristics of each subtype of PLC (Table 1). In the HCC group, we found that the K19^+^ carcinomas presented more vascular invasive characteristics compared to the K19^−^ group (*p* = 0.014). Moreover, cHCC-CCA demonstrated features of a more aggressive behavior such as an increased tumor diameter, vascular invasion, and higher proliferation index (*p* = 0.009, *p* = 0.002, and *p <* 0.001) compared to K19^−^ HCC. Additionally, cHCC-CCA showed a predominance in females, non-viral-infected, and non-cirrhotic livers when compared to HCC K19^−^ (respectively, *p* = 0.006, *p* = 0.007, and *p* < 0.001). Importantly, as part of the clinical work-up, all cHCC-CCA tumors (100%; 35/35) showed positivity for K19 (Figure 1). In the CCA group, no cirrhotic background or viral disease was observed in the corresponding livers. The CCA lesions were more often singular (*p* = 0.008), had a higher proliferation index (*p* ≤ 0.001), and showed prominent vascular invasion (*p* = 0.002) compared to the K19^−^ HCC. This analysis reveals increasing aggressiveness from K19^−^ HCC to K19^+^ HCC, over cHCC-CCA, to CCA. This axis also reflects increasing cholangiocytic characteristics.

### 2.2. Increased Levels of YAP/TAZ as a Defining Marker in cHCC-CCA and CCA versus HCC Patients

To understand the correlation between YAP and TAZ expression and the different subtypes of liver cancer, we evaluated their expression in K19-negative HCCs, K19-positive HCCs, cHCC-CCA, and CCA (Table 1). We observed several cases with only the cytoplasmic presence of YAP and TAZ, while other cases showed a combination of both nuclear and cytoplasmic localization (Figure 1). No cases showed only nuclear expression without the presence of a cytoplasmic staining. A detailed analysis of the cytoplasmic and nuclear presence of YAP and TAZ (Table 1) showed increasing levels of nuclear accumulation along the HCC–CCA axis. In the K19^−^ HCC cases, 81% (66/81) were positive for YAP in the cytoplasm and 64% (52/81) showed nuclear YAP, while 58% (47/81) and 21% (17/81) had cytoplasmic and nuclear TAZ, respectively. The K19^+^ HCC cases showed higher levels of YAP in the cytoplasm (92%) and nucleus (77%) compared to K19-negative cases. Comparable localization patterns were observed for TAZ: K19^+^ HCC and K19^−^ HCC had an almost similar incidence of cytoplasmic TAZ positivity (54% vs. 58%), and a higher incidence of nuclear TAZ positivity in K19^+^ compared to K19^−^ HCC was noted (38% vs. 21%). All cHCC-CCA and CCA showed high levels of TAZ and YAP in the cytoplasm and nucleus. No microscopic differences were observed between the hepatic and cholangiocytic components in cHCC-CCA (Figure 1). 

**Figure 2:**
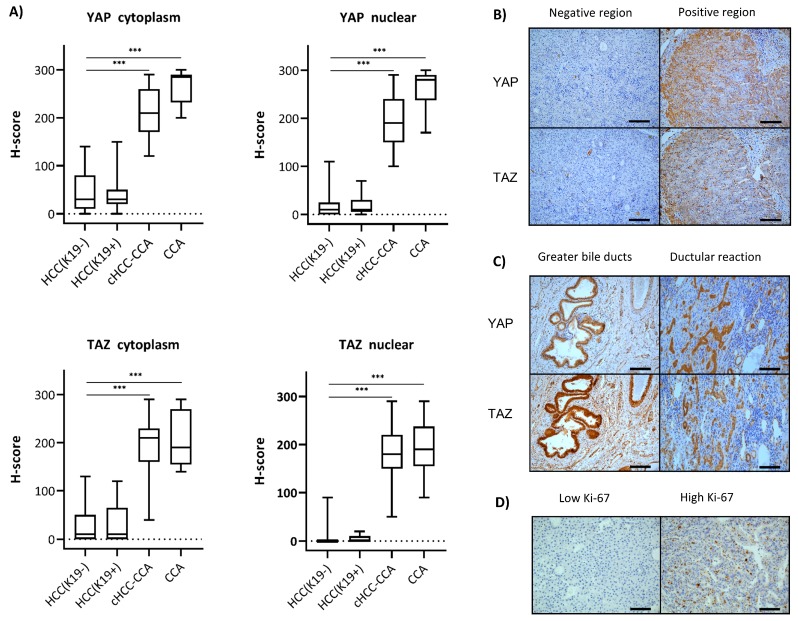
The histoscores (H-scores) of HCC K19^−^, HCC K19^+^, cHCC-CCA, and CCA were plotted (**A**). Statistically significant differences in YAP and TAZ expression in the cytoplasm and nucleus were observed between the HCC groups, cHCC-CCA, and CCA (*** *p* < 0.001). Intratumoral heterogeneity is illustrated in a representative example of an HCC K19- case (**B**). Regions with and without YAP and TAZ were observed in this case. Expression of YAP and TAZ in all compartments of the biliary tree was observed (**C**). Illustration of two HCC cases with a low and high proliferation index (Ki-67) (**D**). All images were taken at 20×. Scale bar: 100 µm.

Notably, the localization pattern of YAP and TAZ was very heterogeneous in multiple cases, especially in the HCC (K19^−^ and ^+^) group; that is, while some parts of the tumor were completely negative, others were strongly positive (Figure 2). Therefore, we used the histoscore (H-score) to evaluate the intensity and area of YAP/TAZ expression in tumor cells (Figure 2). Notably, this analysis revealed that the H-score of YAP and TAZ in the cytoplasm of the HCC groups was significantly lower compared to those of the cHCC-CCA and CCA groups (*p* < 0.001 and *p* < 0.001) (Figure 2). Analogous results were seen when the nuclear presence of YAP and TAZ was scored and compared between HCC and cHCC-CCA (*p* < 0.001 and *p* < 0.001). To conclude, our immunohistochemical analysis showed an increasing YAP/TAZ positivity along the HCC–CCA axis.

### 2.3. Co-Expression Profile of YAP and TAZ

In the K19^−^ HCC group, the cytoplasmic presence of YAP was always together with nuclear positivity (Table 2). In addition, positivity of TAZ in the nucleus of K19^−^ HCC was accompanied by positivity of YAP in the nucleus in 94% (16/17) of the cases. Additionally, we scored the co-expression of YAP and TAZ. We found that 31% (16/52) of the YAP-positive K19^−^ HCCs were also positive for TAZ and, conversely, that 94% (16/17) of TAZ-positive K19^−^ HCCs were also positive for YAP (Table 2). In the K19^+^ HCC group, which was too small to obtain statistically significant results, similar ratios of YAP/TAZ as in the K19^−^ HCC group were observed (Table 2). YAP and TAZ were broadly expressed in all cases of the cHCC-CCA and CCA groups, for both cytoplasmic and nuclear presence (Figure 2 and Table 2).

### 2.4. Correlation of YAP and TAZ Expression with Clinicopathological Parameters

We evaluated the pathological parameters in accordance to YAP and TAZ nuclear localization and found a significant association between a higher proliferation index of the tumor and the nuclear presence of YAP and TAZ (*p <* 0.001 and *p <* 0.001) in the K19^−^ HCC cases (Table 2 and Figure 2). Interestingly, a non-viral background was more often associated with absence of nuclear TAZ (*p* = 0.026). The proliferation index (Ki-67) was significantly higher in YAP-nuclear-positive HCCs (18/52) compared to YAP-nuclear-negative cases, which all had a low proliferative status. Other clinicopathological parameters showed no statistically significant association with YAP or TAZ status. Another pathological observation was strong nuclear and cytoplasmic expression of YAP and TAZ in the ductular reaction, which contains the hepatic progenitor cells (Figure 2). The expression pattern is reminiscent of the high expression in the normal intra- and extrahepatic bile ducts of the biliary tree.

### 2.5. Clinical Relevance of Nuclear and Cytoplasmic Presence of YAP and TAZ

**Figure 3:**
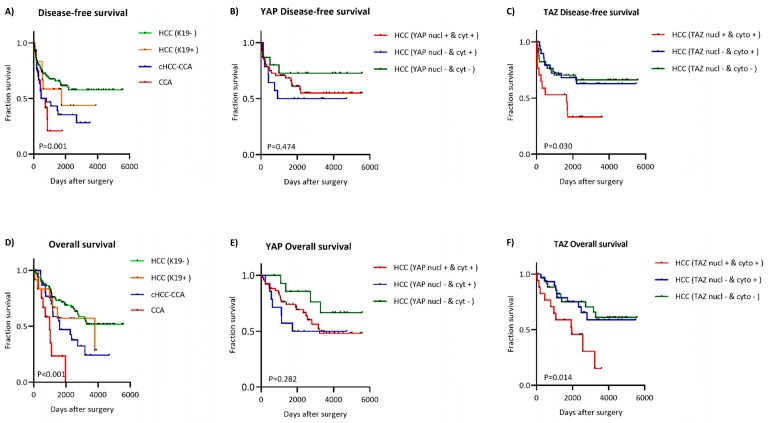
Kaplan–Meier curves for disease-free survival and overall survival are illustrated for the subgroups HCC, cHCC-CCA, and CCA (**A**,**D**). Expression of YAP in the nucleus and/or cytoplasm in Kaplan–Meier curves for OS and DFS in HCC K19^−^ (**B**,**E**). TAZ expression in the nucleus and/or cytoplasm in HCC K19^−^ (**C**,**F**).

To evaluate the prognostic significance of YAP and TAZ expression, we examined the Kaplan–Meier curves for disease-free survival (DFS) and overall survival (OS) of patient cohorts with or without YAP and TAZ in the nucleus and cytoplasm. HCC K19^−^ cases had better DFS and OS compared to K19^+^ cases, while cHCC-CCA had an even worse prognosis compared to the HCC (K19^+/−^) group. CCA patients had the worst prognosis of all included groups (Figure 3). Since all of the cHCC-CCA and CCA patients had high YAP and TAZ expression, their expression status cannot differentiate prognosis for these groups. However, we further evaluated the K19-negative group because this group showed heterogeneous expression of YAP/TAZ. The K19-positive group was too small to be statistically relevant. To appraise the relevance of nuclear versus cytoplasmic localization of YAP and TAZ, we divided the HCC K19^−^ cohort into three subgroups. The first subgroup was cases which had nuclear and cytoplasmic YAP. The second subgroup was the cases that had cytoplasmic but not nuclear YAP. The third subgroup contained the cases without any positive YAP staining in the nucleus or cytoplasm. The same subdivision was made for TAZ expression. As mentioned above, no cases were solely positive in the nucleus. These nuclear-positive cases were always accompanied by cytoplasmic positivity. Disease-free survival and overall survival analysis showed no statistical difference between the three subgroups when YAP was taken into account, although there was a trend towards a less favorable prognosis when YAP was present in the nucleus and/or cytoplasm (Figure 3). On the other hand, positivity for TAZ in the cytoplasm did not seem to influence the DFS or OS. However, cases with nuclear TAZ had significantly poorer DFS and OS of (*p* = 0.006 and *p* = 0.001). 

In the overall univariate analysis of the HCC K19^−^ patients, a histologically observed tumor size greater than 50 mm (*p* < 0.001) and lymphovascular invasion (*p* < 0.001) were significantly related with a greater chance of recurrence and correspondingly less favorable overall survival (Table 3). Univariate analysis demonstrated the prognostic importance of nuclear localization of TAZ for DFS and OS in HCC K19^−^ patients (*p* = 0.011 and *p* = 0.005). In a multivariate analysis, we included the significant factors associated with a poor DFS and OS. To evaluate YAP and TAZ separately, since their presence is strongly correlated, we performed multivariate Cox regression analysis on both (Table 3). These findings were examined in two multivariate Cox regression models. This analysis revealed that the presence of YAP in the cytoplasm was a significant predictor of poor overall survival for HCC K19^−^ patients (*p* = 0.022, HR: 4.395, 95% CI: 1.235–15.637) (Table 3). YAP nuclear positivity revealed a trend but did not turn out to be a statistically significant predictor of poor survival (*p* = 0.063, HR: 2.823, 95% CI: 0.946–8.424). In the second multivariate model, we observed a significant prognostic importance of TAZ for recurrence and overall survival of the patient when TAZ was present in the nucleus (*p* = 0.043 and *p* = 0.009).

## 3. Discussion

Currently, clinical pathologists lack clear and easy-to-use immunohistochemical markers for the pathological diagnosis of cHCC-CCA [8]. This tumor group expresses stemness markers and has morphological hepatocellular as well as cholangiocellular characteristics mixed within the same tumor. The recent consensus of international experts is therefore solely based on hematoxylin and eosin staining (H&E) with respect to the morphological combined characteristics of the tumor. Markers like Hepatocyte Paraffin 1, arginase-1, and canalicular staining of polyclonal carcinoembryonic antigen (pCEA) are used as hepatocellular markers, while Keratin 7 and Keratin 19 are used to identify the cholangiocytic component. Expression of these markers in the corresponding compartment can help the clinical pathologist. However, the expression of these markers can change during carcinogenesis and become difficult to interpret, and there is a lack of a specific marker for cHCC-CCA. We observed high expression of YAP and TAZ in all cHCC-CCA compared to HCC (K19^+/−^). Additionally, no differences in the expression of YAP and TAZ were observed between the hepatic and cholangiocytic components. Our results on YAP expression are supported by similar findings in another study [16]. Therefore, we propose YAP and TAZ as helpful markers for a more accurate diagnosis of cHCC-CCA. 

Primary liver cancer comprises a diverse group of carcinomas. Intertumoral and intratumoral heterogeneity is seen as one of the difficulties in finding an effective therapy. Within the group of HCC, it is widely supported and demonstrated that there is a distinguishable subgroup of HCC, which is categorized as K19^+^ HCC [10]. These K19^+^ HCCs have a hepatocellular morphology and express stemness markers, of which K19 is the best studied and evaluated [10,11,12]. In our study, we observed a slight increase in YAP/TAZ expression in K19^+^ HCC compared to K19^−^ HCC, although the number of K19^+^ HCC cases was too few to draw statistically significant conclusions or to perform a survival analysis. However, it is clear that the K19^+^ group is more aggressive compared to the K19^−^ group. Nevertheless, we currently do not know whether and how tumor aggressiveness is influenced by YAP/TAZ.

The cell of origin for cHCC-CCA is not known, but there is increasing evidence implying that hepatic progenitor cells are the cells of origin of this tumor [6,23]. A stemness origin was recently indicated by a thorough genomic study of cHCC-CCA cases, which revealed that cHCC-CCA has a common cell lineage for the HCC and CCA component [6]. Here we found high YAP and TAZ expression in both components of cHCC-CCA and also in ductular reactions. The ductular compartment is known to harbor hepatic progenitor cells in different human liver diseases. In addition, YAP and TAZ are active in cancer stem cells and are required for their expansion [24,25,26]. Altogether, these results suggest the existence of a hepatic progenitor cell as the cell of origin for cHCC-CCA.

YAP and TAZ are strongly associated with the specification and maturation of cholangiocytes in the mouse. In addition, they are crucial for the integrity and maintenance of ductular cells and for the expansion of this compartment [27,28]. Analogously, YAP/TAZ are also expressed at high levels in the extra- and intrahepatic compartments of the biliary tree in the human liver. Moreover, recent studies in the mouse highlight the importance of the YAP/TAZ–Notch interplay within the liver [29,30]. At the same time, our previous and more recent work showed that the Notch signaling axis is critical for biliary regeneration through mobilization of the hepatic progenitor cells [31,32]. Taken together, these findings suggest that YAP and TAZ expression are crucial for the development or regeneration of the cholangiocytic lineage.

Tissue microarrays are increasingly popular for gene expression studies in cancer samples because they allow the analysis of large numbers of patients on a few slides. However, the use of tissue microarrays is limited by the very small amount of tumor tissue per patient. This restricts the evaluation of tissue heterogeneity in different regions of a tumor and may thus produce a false evaluation. Therefore, we used large tumor samples, which revealed a surprisingly heterogeneous distribution of YAP and TAZ. This may not have been detected on tissue microarrays. Thus, assessing YAP and TAZ expression should be done using whole tissue slides or multiple sampling regions within one tumor, since limited sampling can lead to an undetected positivity. 

Another interesting clinicopathological finding was the association between YAP nuclear positivity and high proliferative index of tumors in the HCC K19^−^ group. YAP and TAZ bind to TEAD transcription factors and direct the expression of downstream genes related to cell growth and cycle progression [33]. However, the prevalence of a high proliferation index in 18 out of 52 K19^−^ HCC cases with nuclear YAP (Table 2) may be seen as counterintuitive since YAP and TAZ are associated with invasiveness. Our univariate analysis of Ki-67 in HCC (Table 3) and previous work of other groups show that Ki-67 positivity has no clear clinical prognostic value in HCC [34,35]. Thus, the biological function in disease progression of the association between YAP and Ki-67 (proliferation) remains unknown.

Although YAP and TAZ seem to be critical for tumorigenesis, the significance of nuclear versus cytoplasmic presence of YAP and TAZ is still poorly understood [36]. Some studies found a nuclear presence of YAP as a prognostic marker, while others observed comparable results for cytoplasmic presence [37,38,39,40]. Until now, no study investigated YAP or TAZ in the cytoplasm and nucleus within one tumor. Our results suggest that YAP overexpression in the cytoplasm and nucleus has an important role in the aggressiveness of HCC. Indeed, a multivariate Cox regression analysis taking into account the cofounding factors revealed that YAP in the cytoplasm had a significant correlation with poor prognosis in HCC K19^−^ patients. A reason why the prognostic role of YAP positivity is only trending in our cohort rather than definitive as indicated in other studies could be because of the more stringent selection of HCCs in our study [16,41]. We excluded all K19^+^ cases, which are known to be more aggressive. A possible explanation for the rather poor prognosis in HCC with only YAP positivity in the cytoplasm compared to YAP in the nucleus and cytoplasm could be due to the inability to detect weak nuclear positivity by immunohistochemistry in these cases. The undetectability of nuclear YAP could explain why HCCs with a poor prognosis were wrongfully included in the group of “YAP only cytoplasmic positive tumors”, which would lead to a worse prognosis in the YAP-cytoplasmic-positive group compared to the YAP-nuclear-positive group. On the other hand, cytoplasmic presence of TAZ does not seem to have an impact on recurrence-free survival or overall survival, while nuclear TAZ is a very strong predictor of poor prognosis for K19^−^ HCC. Since all cHCC-CCA and CCA were highly positive for cytoplasmic and nuclear YAP and TAZ, it was not possible to perform a survival analysis based on the expression of YAP or TAZ in these tumors. In comparison with the other types of primary liver cancer it was clear that cHCC-CCA had an overall worse prognosis compared to HCC. 

In conclusion, we studied the expression profile of YAP and its less studied homolog TAZ. In contrast to previous work, which mainly examined virally induced HCC, our analysis was performed on a Caucasian, non-viral-dominant cohort of PLC patients and shows insight into the clinical relevance of the cellular localization of YAP and TAZ in HCC K19^−^, HCC K19+, cHCC-CCA, and CCA cases.

## 4. Materials and Methods 

### 4.1. Patients and Samples

A retrospective cohort of 141 patients who underwent surgery for primary liver cancer or cholangiocarcinoma at the University Hospitals in Leuven (Belgium) was included in this study. After surgery, the resection specimen was taken and fixed in 6% buffered formalin, and multiple representable sections were taken. The clinicopathological characteristics included the age, sex, background diseases, prognostic follow-up data, the final pathological diagnosis, tumor size of the largest nodule, multiplicity, vascular invasion, and the histological grading of the tumor (well, moderately, poorly). The ethical committee of the University Hospital of Leuven approved this study (S62171, 30/10/2018). An informed consent was not required in accordance with Belgian Law.

### 4.2. Immunohistochemistry

Immunohistochemistry was performed on formalin-fixed paraffin-embedded sections (FFPE) cut at 4 µm. Antigen epitope retrieval was performed using the DAKO PT Link (Dako, Santa Clara, CA, USA) in a citrate buffer (pH 6) or Tris-EDTA buffer (pH 9) according to the manufacturer’s instructions. Blocking of the endogenous peroxidase was achieved by Dual endogenous Enzyme-Blocking Reagent (Dako, Santa Clara, CA, USA). The slides were subsequently incubated for 30 minutes at room temperature with primary antibodies for YAP (1/100; Abcam, Cambridge, UK), TAZ (1/100; Sigma-Aldrich, Saint Louis, MO, USA), K19 (ready-to-use; Dako), and Ki-67 (ready-to-use; Dako). After incubation of the primary antibody, the slides were incubated with a horseradish-peroxidase-labelled Envison Flex system (Dako) for 30 minutes at room temperature. Peroxidase activity was detected with 3.3′-diaminobenzidinen (Dako) as a substrate. All slides were counterstained with Mayer’s haematoxylin and mounted. Negative and positive controls were included.

The immunoreactivity was evaluated by two independent observers (TR and MVH). The histoscore (H-score) was calculated semi-quantitatively for YAP and TAZ. Expression of YAP and TAZ was graded as 0, negative; 1, weak; 2, moderate; or 3, strong; and the percentage of positive tumor cells was scored separately for the cytoplasm and the nucleus. The possible range of expression scores was between 0 and 300. Cytoplasmic K19 expression in greater than 5% of the tumor cells was considered positive. Nuclear Ki-67 staining in more than 5% of the tumor cells in 10 high-power fields was considered high. We dichotomized the positivity of YAP/TAZ when necessary for survival analysis. A cut-off of ≥10% positivity in the tumour cells was considered positive, which is comparable to previous similar studies [16,39]. 

### 4.3. Statistical Analysis

Statistical analyses were performed using SPSS software version 25 (SPSS inc., Chicago, IL, USA) and GraphPad Prism version 8.00 for Windows (GraphPad Software, San Diego, CA, USA). Chi-square test and Fisher’s exact test were used for the analysis of categorical variables. Differences in continuous variables were investigated using the independent Student’s *t*-test and analyses of variance (ANOVA). ANOVA was followed by Tukey’s multiple comparison test. Clinicopathological variables were dichotomized with respect to their relevance. Disease-free survival (DFS) and overall survival (OS) figures were generated by using the Kaplan–Meier method. The Cox proportional hazard model was used to investigate the effects of each parameter on both survivals separately (univariate models) and combined (multivariate models). The Cox proportional method was also used to determine the hazard ratios (HRs) and the respective 95% confidence intervals (CIs). Multiple testing was not performed due to the explorative nature of this experiment. All *p*-values less than 0.05 were considered statistically significant.

## Figures and Tables

**Table 1:** Clinicopathological characteristics.

Characteristics	HCC (K19^−^) (*n* = 81)	HCC (K19^+^) (*n* = 13)	cHCC-CCA (*n* = 35)	CCA (*n* = 12)	HCC (K19^−^) vs. HCC (K19^+^)	HCC (K19^−^) vs. cHCC-CCA	HCC (K19^+^) vs. cHCC-CCA	HCC (K19^−^) vs. CCA
**Age** (mean ± SD), years	61.3 ± 11.7	64.0 ± 8.9	64.5 ± 9.5	61 ± 11.0	0.528	0.21	0.829	0.703
**Sex**					1	0.006	0.193	0.35
Male	58 (72%)	9 (69%)	15 (43%)	7 (58%)				
Female	23 (28%)	4 (31%)	20 (57%)	5 (42%)				
**Etiology**					1	0.007	0.187	0.014
Viral	29 (36%)	4 (31%)	4 (11%)	0 (0%)				
Non-viral *	52 (64%)	9 (69%)	31 (89%)	12 (100%)				
**Tumor diameter (mm)**					0.369	0.002	0.321	0.564
< 50 mm	54 (67%)	7 (54%)	12 (34%)	9 (75%)				
≥ 50 mm	27 (33%)	6 (46%)	23 (66%)	3 (25%)				
**Differentiation grade**					0.469	0.895	0.368	0.905
Well	12 (15%)	1 (8%)	6 (17%)	3 (25%)				
Moderate	50 (62%)	7 (54%)	22 (63%)	7 (58%)				
Poorly	19 (23%)	5 (38%)	7 (20%)	2 (17%)				
**Number of nodules**					0.553	0.008	0.018	0.008
Single	41 (51%)	5 (38%)	27 (77%)	11 (92%)				
Multiple	40 (49%)	8 (62%)	8 (23%)	1 (8%)				
**Vascular invasion**					0.014	0.009	0.469	0.002
Present	36 (44%)	11 (85%)	25 (71%)	11 (92%)				
Absent	45 (56%)	2 (15%)	10 (29%)	1 (8%)				
**Cirrhotic background**					1	<0.001	<0.001	<0.001
Present	58 (72%)	10 (77%)	5 (14%)	0 (0%)				
Absent	23 (28%)	3 (23%)	30 (86%)	12 (100%)				
**Proliferation (Ki67^+^)**					1	<0.001	0.011	<0.001
Low	63 (78%)	10 (77%)	12 (34%)	2 (17%)				
High	18 (22%)	3 (23%)	23 (66%)	10 (83%)				
**YAP cytoplasmic**					0.452	0.005	0.271	N/A
Present	66 (81%)	12 (92%)	35 (100%)	12 (100%)				
Absent	15 (19%)	1 (8%)	0 (0%)	0 (0%)				
**YAP nuclear**					0.531	<0.001	0.017	N/A
Present	52 (64%)	10 (77%)	35 (100%)	12 (100%)				
Absent	29 (36%)	3 (23%)	0 (0%)	0 (0%)				
**TAZ cytoplasmic**					0.773	<0.001	<0.001	N/A
Present	47 (58%)	7 (54%)	35 (100%)	12 (100%)				
Absent	34 (42%)	6 (46%)	0 (0%)	0 (0%)				
**TAZ nuclear**					0.175	<0.001	<0.001	N/A
Present	17 (21%)	5 (38%)	35 (100%)	12 (100%)				
Absent	64 (79%)	8 (62%)	0 (0%)	0 (0%)				

* Non-viral background in HCC (K19^−^) patients was related to alcoholic steatohepatitis (ASH) (*n* = 21), non-alcoholic steatohepatitis (NASH) (*n* = 15), hemochromatosis (*n* = 3), PBC (*n* = 1), glycogen storage disease (*n* = 1), Wilson’s disease (*n* = 1), and cryptogenic (*n* = 10). For HCC (K19^+^) patients this was related to ASH (*n* = 4), NASH (*n* = 4), and hemochromatosis (*n* = 1). For cHCC-CCA patients this was related to ASH (*n* = 2) and Wilson’s disease (*n* = 1), and all other cases had no known chronic liver disease. SD, standard deviation.

**Table 2:** Clinicopathological characteristics in relation to YAP and TAZ expression.

	HCC (K19^−^)				HCC (K19^+^)				cHCC-CCA	CCA
	YAP Nuclear +/− (*n* = 52/29)	*p*	TAZ Nuclear +/− (*n* = 17/64)	*p*	YAP Nuclear +/− (*n* = 10/3)	*p*	TAZ Nuclear +/− (*n* = 4/8)	*p*	YAP or TAZ Nuclear +/− (*n* = 35/0)	YAP or TAZ Nuclear +/− (*n* = 12/0)
**Age (years)**		0.035		0.202		1.0		1.0		
<65	25/7		9/23		3/1		1/3		24/0	7/0
≥65	27/22		8/41		7/2		3/5		11/0	5/0
**Sex**		0.694		0.478		1.0		1.0		
Male	38/20		11/47		7/2		3/5		15/0	7/0
Female	14/9		6/17		3/1		1/3		20/0	5/0
**Etiology**		0.102		0.026		0.203		1.0		
Viral	22/7		10/19		2/2		1/3		4/0	0/0
Non-viral	30/22		7/45		8/1		3/5		31/0	12/0
**Tumor diameter (mm)**		0.743		0.177		0.192		1.0		
<50 mm	18/9		8/19		6/0		2/3		23/0	9/0
≥50 mm	34/20		9/45		4/3		2/5		12/0	3/0
**Differentiation grade**		0.503		0.341		0.70		0.13		
Well	6/6		3/9		0/1		0/1		6/0	3/0
Moderate	34/16		8/42		4/1		1/6		22/0	7/0
Poorly	12/7		6/13		5/3		3/1		7/0	2/0
**Differentiation grade**		0.503		0.341		0.70		0.13		
Well	6/6		3/9		0/1		0/1		6/0	3/0
Moderate	34/16		8/42		4/1		1/6		22/0	7/0
Poorly	12/7		6/13		5/3		3/1		7/0	2/0
**Number of tumor nodules**		0.436		0.064		0.231		0.208		
Single	24/16		5/35		5/3		4/4		8/0	11/0
Multiple	28/13		12/29		5/0		0/4		27/0	1/0
**Vascular invasion**		0.604		0.059		1.0		1.0		
Present	22/14		11/25		8/3		4/7		25/0	11/0
Absent	30/15		6/39		2/0		0/1		10/0	1/0
**Cirrhotic background**		0.251		0.191		0.528		1.0		
Present	35/23		9/49		7/3		3/7		5/0	0/0
Absent	17/6		6/15		3/0		1/1		30/0	12/0
**Proliferation index (Ki67^+^)**		<0.001		0.006		0.528		1.0		
Low	34/29		9/54		7/3		3/6		12/0	2/0
High	18/0		8/10		3/0		1/2		23/0	12/0
**YAP cytoplasmic positivity**		< 0.001		0.027		0.230		1.0		
Present	52/14		17/49		10/2		4/7		35/0	12/0
Absent	0/15		0/15		0/1		0/1		0/0	0/0
**YAP nuclear positivity**	N/A	N/A		0.004	N/A	N/A		1.0	N/A	N/A
Present			16/36				3/6			
Absent			1/28				1/2			
**TAZ cytoplasmic positivity**				<0.001		1.0		0.061		
Present	36/11		17/30		6/1		4/2		35/0	12/0
Absent	16/18		0/34		4/2		0/6		0/0	0/0
**TAZ nuclear positivity**		0.003	N/A	N/A		1.0		N/A	N/A	N/A
Present	16/1				3/1		4/0			
Absent	36/28				6/2		0/8			

**Table 3:** Univariate and multivariate analysis for overall survival and disease-free survival in HCC K19^−^.

Clinicopathological Characteristics		DFS		OS	
	Numbers	HR (95% CI)	*p*	HR (95% CI)	*p*
**Univariate Cox Regression Analysis**					
Sex: male vs. female	58/23	0.996 (0.461–2.154)	0.992	0.952 (0440–2.059)	0.901
Age: ≥65 years vs. <65 years	32/49	1.312 (0.652–2.641)	0.447	1.617 (0.803–3.255)	0.178
Histological differentiation grade: well	12	1		1	
Histological differentiation grade: moderately	50	0.668 (0.242–1.839)	0.435	0.725 (0.261–2.017)	0.538
Histological differentiation grade: poorly	19	1.957 (0.688–5.571)	0.208	2.316 (0.824–6.515)	0.111
Diameter: ≥50 mm vs. <50 mm	54/27	5.794 (2.778-12.086)	<0.001	5.046 (2.428–10.489)	<0.001
Lymphovascular invasion: present vs. absent	36/45	3.534 (1.696–7.364)	0.001	3.867 (1.844–8.110)	<0.001
Multiple noduli: present vs. absent	41/40	1.268 (0.633–2.540)	0.503	1.153 (0.576–2.311)	0.687
Proliferation index: high vs. low	18/63	1.672 (0.773–3.617)	0.192	1.630 (0.753–3.529)	0.215
Viral background: present vs. absent	29/54	0.914 (0.440–1.897)	0.809	1.031 (0.495–2.144)	0.936
YAP cytoplasmic: present vs. absent	66/15	1.748 (0.613–4.985)	0.297	2.133 (0.746–6.102)	0.158
YAP nuclear: present vs. absent	52/29	1.093 (0.527–2.267)	0.811	1.212 (0.583–2.519)	0.606
TAZ cytoplasmic: present vs. absent	47/34	1.496 (0.721–3.104)	0.280	1.654 (0.795–3.444)	0.178
TAZ nuclear: present vs. absent	17/46	2.590 (1.247–5.380)	0.011	2.880 (1.372–6.042)	0.005
**Multivariate Cox regression analysis**					
**Model 1**					
Diameter: ≥50 mm vs. <50 mm		4.709 (2.151–10.307)	<0.001	3.908 (1.759–8.679)	0.001
Lymphovascular invasion: present vs. absent		2.243 (1.019–4.963)	0.045	2.624 (1.165–5.909)	0.020
YAP nuclear^−^ cytoplasm^−^		1		1	
YAP nuclear^−^ cytoplasm^+^		2.622 (0.759–9.054)	0.127	4.395 (1.235–15.637)	0.022
YAP nuclear^+^ cytoplasm^+^		1.705 (0.578–5.028)	0.333	2.823 (0.946–8.424)	0.063
**Model 2**					
Diameter: ≥50 mm vs. <50 mm		4.408 (2.048–9.489)	<0.001	3.787 (1.734–8.272)	0.001
Lymphovascular invasion: present vs. absent		2.604 (1.135–5.974)	0.024	3.027 (1.252–7.321)	0.014
TAZ nuclear^−^ cytoplasm^−^		1		1	
TAZ nuclear^−^ cytoplasm^+^		1.827 (0.717–4.652)	0.207	2.608 (0.971–7.005)	0.057
TAZ nuclear^+^ cytoplasm^+^		2.396 (1.029–5.580)	0.043	3.123 (1.332–7.319)	0.009

DFS, disease free survival; OS, overall survival; HR, hazard ratio; 95% CI, 95% confidence interval.

## Abbreviations

HCC	Hepatocellular carcinoma
PLC	Primary liver cancer
HBV	Hepatitis B virus
HCV	Hepatitis C virus
NAFLD	Non-alcoholic fatty liver disease
cHCC-CCA	Combined hepatocellular–cholangiocarcinoma
CCA	Cholangiocarcinoma
K19	Keratin 19
K19^+^	Keratin 19 positive
K19^−^	Keratin 19 negative
YAP	Yes-associated protein 1
WWTR1	WW domain-containing transcription regulator protein 1
TAZ	Transcriptional co-activator with PDZ-binding motif
MST1/2	STE20-like protein kinases 1/2
LATS1/2	Large tumor suppressor 1/2
Sav1	Salvador 1
MOB1A/B	Mob kinase activator 1A
DFS	Disease-free survival
OS	Overall survival
ASH	Alcoholic steatohepatitis
NASH	Nonalcoholic steatohepatitis
PBC	Primary biliary cholangitis
